# Review of the Manitoba cohort of patients with hereditary angioedema with normal C1 inhibitor

**DOI:** 10.1186/s13223-019-0381-y

**Published:** 2019-11-12

**Authors:** Lundy McKibbin, Colin Barber, Chrystyna Kalicinsky, Richard Warrington

**Affiliations:** Section of Allergy and Clinical Immunology, Department of Internal Medicine, Rady Faculty of Medicine, University of Manitoba, 820 Sherbrook St, Gc319, Winnipeg, MB R3A 1R9 Canada

**Keywords:** Hereditary angioedema with normal C1 inhibitor, Type 3 hereditary angioedema, Icatibant

## Abstract

**Background:**

Hereditary angioedema with normal C1 inhibitor (HAE-nC1 INH) is a rare, underappreciated condition characterized by recurrent subcutaneous angioedema. The underlying pathophysiology and diagnostic criteria continues to evolve. There is a significant overlap between HAE-nC1 INH and idiopathic nonhistaminergic angioedema, ultimately this may be found to be the same condition. Characterization of cohorts suspected to have either of these conditions is warranted to help refine diagnosis, pathophysiology, and treatment response.

**Methods:**

A retrospective chart review of 418 patients diagnosed with angioedema was conducted. The following inclusion criteria was used: lack of response to antihistamines, steroids, and epinephrine; normal C4, C1 inhibitor (C1 INH) level and function; lack of urticaria or pruritus; occurrence without offending drugs; and positive family history. Enzyme immunoassays for C1 INH function were performed at the Mayo Clinic. Charts meeting these criteria were reviewed for frequency and type of episodes as well as use and response to therapies.

**Results:**

6 patients met the above criteria. 3 of these completed genetic testing, none were found to have factor XII abnormalities. None had angiopoietin 1 or plasminogen gene sequencing. 5 of 6 patients were successfully treated with C1 INH or tranexamic acid for acute treatment of attacks (4 with C1 INH and 1 with tranexamic acid). 4 patients have used Icatibant with good response (typically under 40 min for near full recovery); of these, 3 required Icatibant as acute treatment after other therapies (C1 inhibitor and tranexamic acid) were ineffective. There were 9 patients who otherwise met criteria, but due to a lack of family history were classified as having idiopathic non-histaminergic angioedema.

**Conclusions:**

This retrospective chart review found 6 HAE-nC1 INH patients in Manitoba. 1 responded to tranexamic acid and not C1 INH, 4 typically responded to C1 INH, and 1 responded exclusively to Icatibant. All patients—4 total—who used Icatibant responded; of these 4 patients, 3 required Icatibant after other therapies had failed.

## Background

Hereditary angioedema with normal C1 inhibitor (HAE-nC1 INH) is a rare, underappreciated condition characterized by recurrent angioedema and can be life threatening if it involves the upper airway. Whereas types I and II Hereditary angioedema result from SERPING1 mutations and are associated with low C1 esterase inhibitor level or function; HAE-nC1 INH, as its name implies, has normal C1 esterase inhibitor studies and appears to be formed by a heterogenous group [4]. There are now at least four identified types of HAE-nC1 INH: those caused by mutations of exon 9 of the factor XII gene, the angiopoietin 1 gene, the plasminogen gene, and those of unknown etiology [4]. Since the discovery of HAE-nC1 INH in 2000, our understanding of the underlying pathophysiology, classification, and diagnostic criteria continues to evolve.

As no laboratory test is available to confirm the presence of HAE-nC1 INH, a high degree of suspicion is required. Clinical decision making requires compatible symptoms in the context of a normal C1 INH level and function and the exclusion of alternative etiologies. Factors that suggest HAE-nC1 INH include: lack of response to epinephrine, antihistamines, and steroids; absence of associated urticaria or pruritus; and an absence of other inciting factors such as drugs, e.g. angiotensin converting enzyme inhibitors [2].

Although family history is extremely useful when making the diagnosis, the rate of de novo mutations in HAE-nC1 INH is unknown; as a reference, 25% of patients with HAE types I and II have de novo mutations, i.e. no family history [3]. Genetic testing is only useful when positive, as only 20–25% of HAE-nC1 INH patients have a FXII mutation [1]. As pathogenic mutations continue to be discovered, genome sequencing may play an increasing role in the diagnosis of HAE-nC1 INH. There is significant overlap between HAE-nC1 INH and idiopathic nonhistaminergic angioedema: ultimately these may be found to be the same condition.

Evidently, our understanding of HAE-nC1 INH requires further elucidation. Characterization of cohorts suspected to have these conditions is warranted to help refine our understanding of diagnosis, pathophysiology, genetic underpinnings, and treatment response in HAE-nC1 INH. Prior to this study, the number of patients with HAE-nC1 INH in Manitoba had not been known, nor had their characteristics been formally analyzed individually or as a group. This cohort’s response to therapy is of special interest as access to certain medications is well established—e.g. C1 inhibitor (C1 INH) and tranexamic acid (TXA)—whereas other medications, such as Icatibant, are restricted to patients with types I and II HAE. In Manitoba, Icatibant is only available to patients with HAE-nC1 INH through expensive visits to emergency departments requiring and contributing to long wait times. Currently no patients with HAE-nC1 INH in Manitoba have qualified for home access to Icatibant. It is, therefore, of interest to note whether Icatibant has shown benefit in this cohort.

## Methods

Ethics approval was received through the University of Manitoba’s Research Ethics Board (HS21713) in addition to approval from the Health Sciences Centre Research Impact Protocol Officer.

**Fig. 1:**
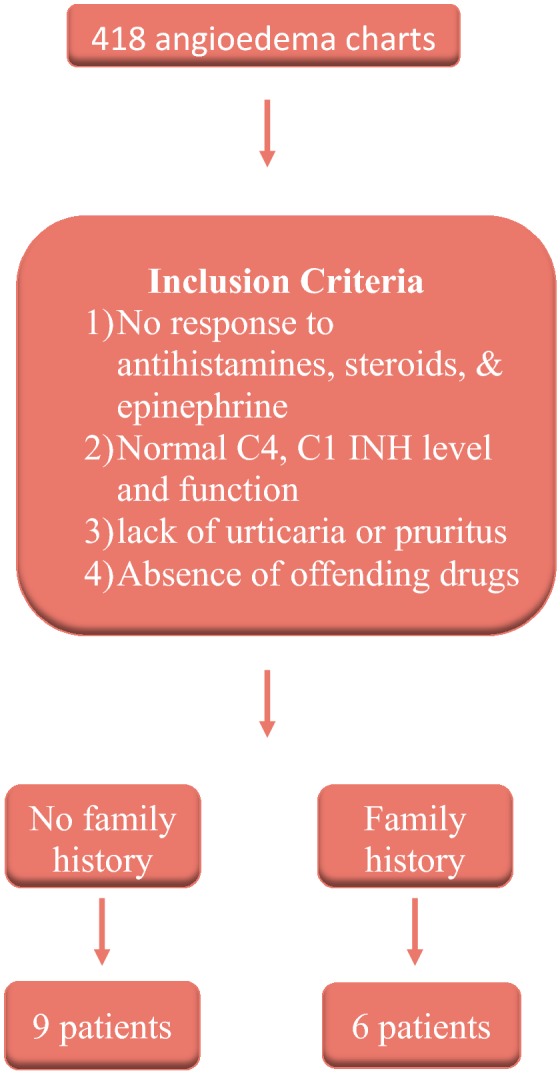
Review of the Manitoba cohort of patients with HAE-nC1 INH: inclusion criteria

A retrospective chart review of 418 charts of patients diagnosed with angioedema was conducted. These charts were first identified by searching a computerized record of all dictated letters from the year 2000 to the present involving patients seen at the Clinical Immunology and Allergy Clinic at the Health Sciences Centre and Grace Hospital in Winnipeg who had a diagnosis of angioedema. As depicted in Fig. 1, the above-mentioned charts were analyzed for the following inclusion criteria: lack of response to antihistamines, steroids, and epinephrine; normal C4, C1 INH level and function; lack of urticaria or pruritus; occurrence without offending drugs; and presence of family history. 6 patients met all inclusion criteria. All available C4 and C1 inhibitor studies were required to be normal; in some cases, these included multiple studies and studies performed during acute episodes. Of note, 9 patients met all criteria except the presence of a family history of angioedema and were not included; the exclusion of patients without a family history of angioedema is in accord with expert opinion and preceding studies, though this is controversial given that de novo mutations and differences in penetrance likely exist [2]. Charts meeting all criteria were reviewed for duration of symptoms, organs involved, and response to therapies.

## Results

6 patients met the above criteria. 3 patients received genetic testing; of the 3 tested, none were found to have factor XII abnormalities. None had angiopoietin 1 or plasminogen gene sequencing. Genetic testing was confined to factor XII and was only performed in 3 patients due to limited availability in our current healthcare system. 4 of 6 patients have used Icatibant and all demonstrated a response. 3 patients responded to Icatibant when other treatments (C1 inhibitor and tranexamic acid) were ineffective. The results of this study are summarized in Table 1.

**Table 1:** Characteristics of patients with HAE-nC1 INH in Manitoba, Canada and their responses to interventions

Patient	Age	Sex	Site(s) of angioedema	C1 INH^a^	TXA^a^	Icatibant^a^	OCP discontinuation
1	60	M	Larynx, tongue	> 6 h	> 8 h	45 min	N/A
2	22	F	Abdomen	40 min	N/A	N/A	Ineffective
3	54	F	Larynx, tongue Extremities Abdomen	30 min	N/A	20 min (given after throat swelling unresponsive to C1 INH)	N/A
4	21	F	Tongue Face Abdomen	Not effective	Usually effective	40 min (after 10 h without benefit post TXA)	↓ Abdominal episodes
5	19	F	Larynx Abdomen	Effective	Effective	Effective	N/A
6	52	F	Extremities	Effective	N/A	N/A	↓ Frequency

*N/A* not applicable; *OCP* oral contraceptive pill

^a^Time to improvement of angioedema symptoms; ↓ decreased; effective/not effective—time not indicated on chart

Patient 1 is a 60-year-old male who lives in rural Manitoba and travels for extended periods—away from medical care—as a long-haul truck driver. He typically has symptoms of laryngeal and tongue angioedema lasting over 24 h without treatment. These symptoms respond to Icatibant within 45 min, to C1 INH within 6–24 h and improvement with tranexamic acid occurs within 8–13.5 h.

Patient 2 is a 22-year-old female with recurrent abdominal symptoms lasting 3 days without treatment. These symptoms respond to as needed C1 INH within 30–40 min; she also uses scheduled C1 INH. Discontinuation of oral contraceptive was of no benefit. She has not tried any other medical treatments. She is a full-time student and has needed to take time away from school due to bothersome headaches and fatigue that occur as side effects of C1 INH therapy.

Patient 3 is a 54-year-old female with symptoms that range from laryngeal and tongue to abdominal and extremity angioedema that last 2–3 days without treatment. Episodes typically improve within 20–30 min after use of as needed C1 INH; however, she did have recurrence of laryngeal edema on one occasion post 1500 units of Berinert that required Icatibant for symptom resolution. She began responding to Icatibant within 20 min. She also uses scheduled C1 INH.

Patient 4 is a 21-year-old female with recurrent tongue, facial, and abdominal angioedema. She has noted benefit with discontinuation of oral contraceptives. She has discontinued scheduled and as-needed C1 INH due to lack of response, but typically responds to tranexamic acid. She did, however, have one 10 h episode of facial angioedema that did not respond to 2 doses of tranexamic acid. She then received Icatibant which resulted in resolution of her symptoms within 40 min.

Patient 5 is a 19-year-old female with laryngeal and abdominal symptoms that have responded well to as-needed C1 INH, tranexamic acid, and Icatibant. She has only had laryngeal edema on one occasion (which responded to a single dose of C1 INH).

Patient 6 is a 52-year-old female with recurrent angioedema of her extremities that has significantly improved after discontinuation of oral contraceptives. She finds as-needed C1 INH effective for her symptoms. She has not tried any other therapies.

## Discussion

This retrospective chart review found 6 HAE-nC1 INH patients in Manitoba: 1 male and 5 females. Although small in number, this cohort’s female predominance is in keeping with the reported male-to-female ratio of 1:6 in patients diagnosed as HAE-nC1 INH of unknown cause [1]. According to this review and an estimated Manitoba population of 1.3 million, the prevalence of HAE-nC1 INH in Manitoba is approximately 5 per million people. Unfortunately, the general prevalence of HAE-nC1 INH is not known, so this cannot be used for comparison at present. 3 of 6 had factor XII testing, with no abnormalities identified; none had angiopoietin 1 or plasminogen gene testing.

2 of 3 patients who discontinued oral contraceptives found discontinuation beneficial. 3 patients received tranexamic acid. 2 patients usually responded to tranexamic acid; with one using tranexamic acid as the mainstay of her therapy, as she did not respond to C1 INH. Additionally, 4 of 6 patients usually responded to C1 INH for acute episodes.

Lastly, 4 of 6 patients received Icatibant; of the 4, 3 used Icatibant after other therapies had failed. 2 of these patients have had life-threatening symptoms that resolved only with Icatibant. Of note, one of these patients is a long-haul truck driver and spends most of his time in remote locations, distant from acute medical care facilities. This suggests that access to Icatibant as an out patient would be of great benefit to a select few patients.

There are many shortcomings of this series, these include a high probability of currently undiagnosed patients, the incomplete documentation of charts, a lack of complete genetic testing, and an incomplete understanding of HAE-nC1 INH. As time passes and diagnostic criteria evolve, it will be interesting to note if the 9 patients who otherwise met criteria, but due to a lack of family history were classified as having idiopathic non-histaminergic angioedema, will eventually be diagnosed with HAE-nC1 INH. One future direction would be to study this group, as its constituents have presentations typical of HAE-nC1 INH despite a lack of family history. None of these 9 patients excluded on the basis of an absent family history have received genetic testing; however, 2 of the 9 have plans to pursue genetic testing privately, as testing is not readily available through our current healthcare system. It would be interesting to see if known genetic abnormalities can explain some or all of these patients’ clinical presentations.

Other future directions include establishing a Manitoban database for patients with hereditary angioedema, which is now underway; collaborating with geneticists for additional testing; and advocating for Icatibant coverage through provincial Pharmacare. There is much to learn about HAE-nC1 INH and this review of the Manitoba cohort of patients with HAE-nC1 INH demonstrates the need for greater understanding.

## Conclusions

This retrospective study found 1 male and 5 females in Manitoba meeting the above-mentioned criteria for HAE-nC1 INH. Several therapies—including contraceptive discontinuation, tranexamic acid, C1 INH, and Icatibant—were noted to be beneficial; however, responses were variable among the cohort. Of the 4 that used Icatibant for episodes of acute angioedema, all found it effective. Interestingly, 3 found Icatibant effective after other therapies had failed, and 2 of these patients had potentially life-threatening symptoms that did not respond to other therapies. There remains much to be learned about this condition, including ideal management and the need for clear diagnostic criteria that might permit these patients to have regular access to Icatibant when effective, as is the situation with HAE Types 1 and 2.

## Data Availability

The data generated during and/or analysed during the current study are not publicly available due to interests of patient confidentiality. Please contact the author for data requests.

## Abbreviations

C1 INH: C1 Inhibitor
HAE-nC1 INH: hereditary angioedema with normal C1 inhibitor
N/A: not applicable
OCP: oral contraceptive pill
TXA: tranexamic acid
